# Comparison of Outcomes Between Functionally and Mechanically Aligned Total Knee Arthroplasty: Analysis of Parallelism to the Ground and Weight-Bearing Position of the Knee Using Hip-to-Calcaneus Radiographs

**DOI:** 10.3390/jpm15030091

**Published:** 2025-02-27

**Authors:** Hongyeol Yang, Chanjin Park, Jaehyeok Cheon, Jaeyeon Hwang, Jongkeun Seon

**Affiliations:** Department of Orthopaedic Surgery, Chonnam National University Medical School and Hospital, 322, Seoyang-ro, Hwasun 58128, Republic of Korea; stephano.h.yang@gmail.com (H.Y.); good6621@naver.com (C.P.); cjh5904@gmail.com (J.C.); hjy5139@naver.com (J.H.)

**Keywords:** total knee arthroplasty, functional alignment, parallelism, joint line orientation, clinical outcomes, ground mechanical axis

## Abstract

**Background:** The objective of this study was to compare the outcomes between patients undergoing mechanically aligned conventional total knee arthroplasty (MA-CTKA) and functionally aligned robotic-arm-assisted TKA (FA-RTKA). **Methods:** We reviewed a prospectively collected database of consecutive patients who underwent primary total knee arthroplasty (TKA) for knee osteoarthritis between June 2022 and May 2023. Patients were divided into two groups—MA-CTKA (n = 50) and FA-RTKA (n = 50)—based on the introduction of a robotic-arm-assisted system during the study period. The hip–knee–ankle (HKA) angle, joint line orientation angle (JLOA) relative to the floor, and weight-bearing line (WBL) ratio were evaluated using full-length standing hip-to-calcaneus radiographs to compare the conventional mechanical axis (MA) and the ground mechanical axis (GA) passing through the knee joint between the groups. Clinical outcomes were also compared between the two groups. **Results:** There were no significant differences in the postoperative HKA angle between the groups, due to discrepancies in the targeted alignment strategies (FA-RTKA: 2.0° vs. MA-CTKA: 0.5°; *p* = 0.001). The postoperative JLOA in the FA-RTKA group was more parallel to the floor, whereas the MA-CTKA group showed a downward angulation toward the lateral side (0.6° vs. −2.7°; *p* < 0.001). In the FA-RTKA group, the GA passed through a neutral position when accounting for the calcaneus, while the MA-CTKA group showed a more lateral GA position (48.8% vs. 53.8%; *p* = 0.001). No significant differences in clinical outcomes were shown between the FA-RTKA and MA-CTKA groups, with the FA-RTKA group demonstrating higher Forgotten Joint Scores and a greater range of motion (all *p* < 0.05). **Conclusions:** Functionally aligned TKA demonstrated improved joint line parallelism to the floor and more neutral weight-bearing alignment in the GA compared to mechanically aligned TKA. These findings indicate a more balanced load distribution across the knee, which may contribute to the superior clinical outcomes observed in the functionally aligned group.

## 1. Introduction

Restoration of a neutral mechanical axis (MA) has traditionally been the goal of successful and reliable total knee arthroplasty (TKA) [1,2]. While mechanically aligned TKAs have shown favorable outcomes, this approach alters the joint line orientation relative to the patient’s native anatomy [3,4]. Notably, approximately 20% of patients report dissatisfaction following TKA [5,6,7]. To address these issues, including patient dissatisfaction and altered joint line orientation, a shift towards a more “patient-specific” alignment philosophy in TKA has emerged recently [8,9].

The concept of anatomic restoration has garnered increasing attention, particularly in the context of kinematically aligned TKA. This approach seeks to maintain the pre-arthritic alignment of the limb and joint line based on the individual’s unique constitutional anatomy [4,10,11,12]. In response, hybrid alignment techniques, which integrate kinematic alignment within a safe range of alignment and balanced soft tissues, have been explored. These techniques have demonstrated satisfactory clinical outcomes in numerous studies, with advancements in robotic arm-assisted methods [13,14,15,16,17,18,19,20]. In particular, the evolution of surgical technology has led to the development of robotic-arm-assisted TKA, and functional alignment aims to restore the native joint plane, while prioritizing soft tissue balance as the primary goal of alignment [21,22,23,24,25].

The ground mechanical axis (GA), defined as the hip-to-calcaneus axis, ideally passes through the center of the knee joint in the native knee and has gained attention as a potential alternative to the hip-to-ankle axis for alignment evaluation [26,27,28]. Haraguchi et al. proposed assessing the true mechanical axis (MA) from the femoral head center to the ground reaction point at the lowest aspect of the calcaneus rather than at the ankle [28]. Victor et al. emphasized that in the natural knee during bipedal stance, the joint line is generally parallel to the ground. They underscored the importance of evaluating postoperative joint line parallelism to the floor, as this alignment optimizes load distribution and minimizes shear stress [3,29]. However, only a few previous reports have evaluated the joint line orientation and weight-bearing positions of the knee by comparing different alignment methodologies [3,30,31].

To address this gap, the aims of the present study were to compare (1) the joint line parallelism to the floor and GA ratios of the knee joint under true conditions, accounting for the calcaneus, between patients undergoing mechanically aligned conventional TKA (MA-CTKA) and functionally aligned robotic-arm-assisted TKA (FA-RTKA), and compare (2) postoperative clinical outcomes between these two methods of TKA. Our hypothesis was that functionally aligned TKA would more efficiently achieve a more parallel joint line orientation to the floor with a more neutral weight-bearing in the GA and thus eventually lead to better PROMs than mechanically aligned TKA.

## 2. Materials and Methods

### 2.1. Patients

This study was approved by the Institutional Review Board of Chonnam National University Hwasun Hospital (No. CNUHH-2024-024); approval date 15 February 2024). We retrospectively reviewed a prospectively collected database, which included 155 patients who underwent TKA with the Triathlon prosthesis (Stryker, Mahwah, NJ, USA) for knee osteoarthritis between June 2022 and May 2023. Patients were divided into two groups based on the timing of their surgery relative to the introduction of the robotic-arm-assisted system (Mako, Stryker, MI, USA) in our department. For those undergoing robotic-arm-assisted TKA, a restricted functional alignment (FA) workflow was utilized, with a 3-degree varus coronal limb and tibial component alignment restriction [32,33]. The inclusion criteria were patients who underwent either FA-RTKA or MA-CTKA for knee osteoarthritis and were evaluated using the full-length standing hip-to-calcaneus radiographs. Exclusion criteria included a history of ipsilateral knee surgery, inflammatory arthritis, posttraumatic osteoarthritis, or severe arthritic bone loss. A total of 100 patients met the criteria and were included in the final analysis, with 50 patients in the FA-RTKA and 50 in the MA-CTKA group.

### 2.2. Surgical Techniques

All surgeries were performed in a standard manner by a single, highly experienced surgeon using the posterior-stabilized (PS) Triathlon system (Stryker, Mahwah, NJ, USA). A discrepancy in the principles underlying the targeted alignment strategies was shown between the two groups (Table 1).

#### 2.2.1. Mechanically Aligned TKA Using a Conventional Jig

Conventional TKAs were conducted using the modified gap technique with the objective of achieving neutral mechanical alignment. Surgeries were performed with conventional instruments and alignment jigs, including an intramedullary femoral jig and an extramedullary tibial jig, to guide bone resection. Medial soft tissue release was performed via subperiosteal elevation of the tibial sleeve. Tibial resections were initiated using an extramedullary guide system with a posterior slope of 3°, while distal femoral resections were conducted using an intramedullary guide, with the valgus angle adjusted to 4–6° based on individual femoral anatomy. Following the proximal tibial and distal femoral resections, soft tissue releases were performed to achieve neutral limb alignment and a symmetrical extension gap. The femoral resection line was marked parallel to the tibial cut at 90° flexion under distraction applied with a laminar spreader and manual tension. After evaluating stability in both extension and 90° of flexion, the surgeon implanted the final components using cement.

#### 2.2.2. Functionally Aligned TKA Using the Robotic-Arm-Assisted System

**Figure 1:**
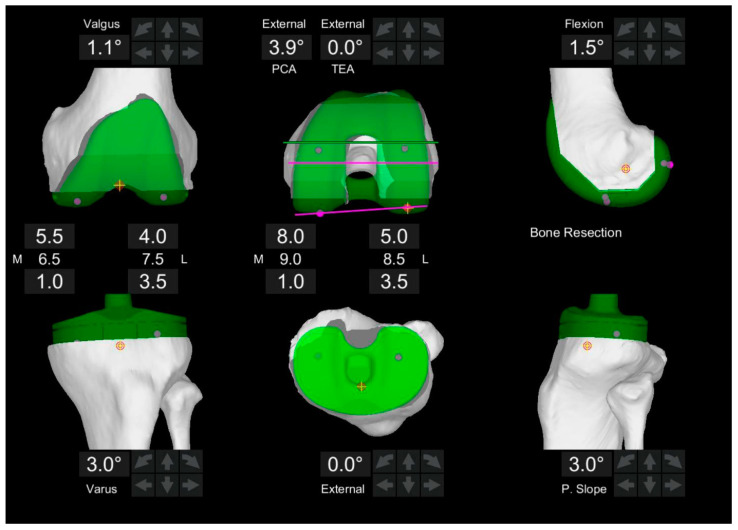
Intraoperative component position using the functional alignment principle. The tibial component alignment was first set to 3.0° (varus) in a coronal alignment to maintain the native tibial joint line within the boundaries; subsequently, the extension and flexion gaps were balanced by fine-tuning the femoral component alignment in all three dimensions. The femoral component coronal alignment was set to −1.1° (valgus) and 3.9° externally rotated relative to the posterior condylar axis. The symbols represent the same reference point.

Mako software 1.0 analyzed preoperative computed tomography (CT) scans to create a patient-specific three-dimensional knee model, enabling precise virtual implant positioning during surgical planning. Guided by the principles of restricted functional positioning, modifications were made to limb alignment, bone resection depths, and implant sizing. Following the registration and validation of bony landmarks, soft tissue balance was assessed in the planned component position by applying dynamic stress to the knee. This evaluation aimed to optimize maximal gap symmetry, with gaps measured medially and laterally in extension (approximately 10° to relieve posterior capsule tension) and in flexion (90°). Based on these assessments, adjustments to the component positioning were made in accordance with restricted FA principles until balance was achieved, or alignment boundaries were reached. Following proximal tibial cuts, a tibial trial implant was positioned, and a second dynamic varus and valgus stress test was conducted to reassess knee kinematics and soft tissue balance. The femoral component was further adjusted to optimize gap balancing (Figure 1). Gap balance was considered achieved with equal medial gaps of 18−19 mm in extension and flexion, with an acceptable slight lateral laxity of 1−2 mm. If residual fixed deformities remained, additional ligamentous or capsular releases were performed within the component alignment boundaries. All remaining femoral bone cuts were completed, and trial implants were inserted. The final insert thickness was determined based on the surgeon’s evaluation of optimal laxity.

### 2.3. Radiographic Assessment

The hip–knee–ankle (HKA) angle, medial proximal tibial angle (MPTA), lateral distal femoral angle (LDFA), and joint line orientation angle (JLOA), were measured during the preoperative radiographic evaluation. The HKA angle was defined as the angle formed by the MA of the femur and tibia, with positive values indicating varus alignment. The MPTA was determined as the angle between the tibial mechanical axis and the joint line of the proximal tibia on the medial side, whereas the LDFA represented the angle between the femoral mechanical axis and the joint line of the distal femur on the lateral side. The JLOA was measured as the angle between a line drawn along the proximal tibial joint surface and a line parallel to the floor, with positive values indicating a tibial joint line angled downward toward the medial side.

Postoperative radiographic evaluations were performed to compare the effects of MA-CTKA and FA-RTKA on lower limb alignment. We measured the HKA angle, JLOA relative to the floor [3,34], and two distinct mechanical axes: the traditional MA (MA: line from the center of the femoral head to the center of the talus) and “true” MA (GA: line from the center of the femoral head to the lowest point of the calcaneus). When measuring the weight-bearing position in the knee joint, the WBL ratio was calculated as the two axes (GA and MA) passing through tibial plafond (i.e., medial edge as 0% and lateral edge as 100%). These comparisons highlight the fundamental differences in weight-bearing distribution and joint line positioning between the two approaches.

### 2.4. Clinical Evaluation

The clinical assessment included patient-reported outcome measures (PROMs) such as the Knee Injury and Osteoarthritis Outcome Score (KOOS) [35] and the Forgotten Joint Score (FJS), evaluated preoperatively and at 1-year postoperatively [36]. Additionally, range of motion (ROM) was measured using a goniometer at the same time points. All clinical outcomes were assessed by two independent investigators who were blinded to the surgical procedures and radiographic analyses.

### 2.5. Statistical Analysis

Normally distributed variables were analyzed using paired and independent *t*-tests, while non-normally distributed variables were assessed with the Wilcoxon signed-rank test and Mann–Whitney U test. Categorical variables were analyzed using the chi-square test or Fisher’s exact test. A *p*-value of < 0.05 was considered statistically significant.

## 3. Results

A total of 100 patients were included in this study, with 50 patients undergoing FA-RTKA and 50 patients receiving MA-CTKA. As shown in Table 2, no significant differences were observed in the demographic characteristics or clinical data between the two groups (Table 2).

### 3.1. Radiological Outcomes

**Figure 2:**
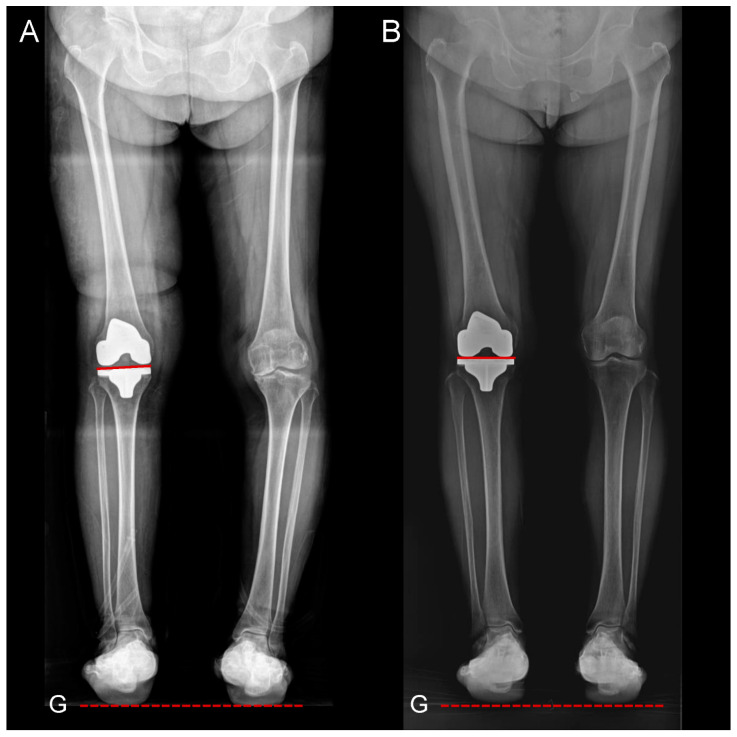
Assessment of the JLOA on hip-to-calcaneus radiographs. (**A**) In the functionally aligned group, the postoperative JLOA was parallel to the ground. (**B**) In the mechanically aligned group, the postoperative JLOA was inclined downward toward the lateral side. The dotted line indicates the ground orientation (G). The JLOA was defined as the angle between the proximal tibial joint surface (solid red line) and the ground (dashed red line).

As shown in Table 3, significant differences were observed between the groups in postoperative HKA angle and component alignment. The FA-RTKA group exhibited greater deviations from neutral coronal alignment, including increased tibial varus and femoral valgus (all *p* < 0.05). When the postoperative JLOA was compared, the FA-RTKA group showed a more parallel joint orientation in relation to the floor (0.6° ± 1.4°), while the JLOA in the MA-CTKA group angled down toward the lateral side (−2.7° ± 2.1°; *p* < 0.001) (Figure 2).

**Figure 3:**
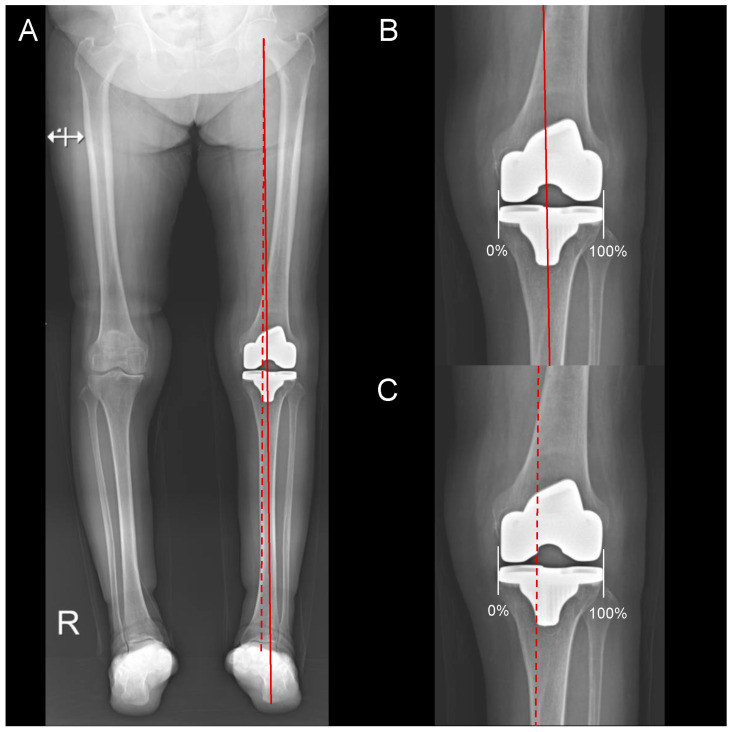
Assessment of weight-bearing position in the knee joint on hip-to-calcaneus radiographs in functionally aligned total knee arthroplasty. (**A**) The Ground Mechanical Axis, defined as the line (GA; solid red) extending from the center of the femoral head to the lowest point of the calcaneus, runs lateral to the traditional mechanical axis (MA; dashed red line). (**B**) Under the “true” condition, when accounting for the calcaneus, the GA passes through the center of the knee joint. (**C**) In contrast, the traditional MA passes slightly medially through the knee joint. The ‘R’ symbol represents ‘Right’ in the X-ray marking.

Significant differences were observed in the weight-bearing position of the knee joint between the two groups, with the mean GA ratios being 48.8% ± 7.0% in the FA-RTKA group and 53.8% ± 8.8% in the MA-CTKA group (*p* = 0.002). The points where the GA passed through the knee joint were more lateral than those where traditional MA passed; thus, the FA-RTKA group had more neutral weight-bearing than the MA-CTKA group when considering the calcaneus (Figure 3).

### 3.2. Clinical Outcomes

PROMs are presented in Table 4. The FA-RTKA group showed higher FJS scores and greater range of motion following TKA than the MA-CTKA group at the 1-year follow-up (all *p* < 0.05).

## 4. Discussion

The main findings of the present study were that functionally aligned TKA more efficiently achieved a parallel joint configuration of the knee relative to the floor with more neutral weight-bearing in the GA compared to mechanically aligned TKA. These differences indicate that the functionally aligned group exhibited a more balanced load distribution around the knee, potentially contributing to a more “natural feeling” in the joint. This observation aligns with findings from previously reported clinical and biomechanical studies [30,37,38,39].

Recent studies have identified foot and ankle joint conditions as important factors related to lower limb alignment that can increase the MA deviation around the knee joint [40,41]. The ankle and hindfoot influence how load is transmitted through the knee joint, which can potentially affect the performance and survival of TKA components [42]. Furthermore, because the calcaneus is considered to be the weight-bearing bone of the lower extremity that transfers body weight to the ground, we used hip-to-calcaneus radiographs in our study series because we believe it to be essential to determine the proper estimation of the optimal knee alignment in parallel to the ground and the “true” MA of the entire lower limb when assessing or planning for TKA.

The debate regarding leg orientation and alignment technique in TKA remains unresolved. There are increasingly divergent views emerging that question the direct association between postoperative coronal misalignment and prosthesis longevity [43,44]. Kinematic alignment is a contemporary approach focused on restoring the pre-arthritic coronal axis that aims to closely restore the anatomic knee phenotype to its constitutional state for each selected patient [45]. Functional alignment, an evolution of the kinematic alignment approach, seeks to maintain the joint plane while achieving balanced gaps in configurations that minimally compromise the periarticular soft tissue envelope [21,22,23,24,25]. In this study, a robotic system was employed to mitigate the risk of significant varus tibial malalignment and to ensure that overall limb alignment remained within the safe zone of 0° ± 3° of coronal alignment. This study highlights the potential advantage of incorporating robotic systems in TKA to achieve more reliable and reproducible surgical outcomes

Bellemans et al. reported that 17% of asymptomatic patients have a constitutional alignment of 3° varus or more, and their findings suggested that neutral alignment following TKA would be unnatural for individual patients and alter their natural anatomy in a substantial proportion of the population [17]. Another study showed that the joint line is usually parallel to the floor in the bipedal stance phase of the native knee [3]. Given the proposed benefits of improved joint line parallelism in relation to the ground, several authors have recently demonstrated the influence of postoperative measurement of the JLOA on clinical outcomes after TKA [3,30,46,47]. In this study, joint line parallelism was used as a measurable outcome to evaluate the effectiveness of functional alignment compared to mechanical alignment. In theory, functional alignment aims to restore the native joint plane while minimizing soft tissue release; consequently, knee joint kinematics and the joint line configuration should be restored [48,49]. The functionally aligned group demonstrated a postoperative JLOA that was more parallel to the floor (0.6° ± 1.7°) and perpendicular to the weight-bearing axis. In contrast, the mechanically aligned group showed a postoperative JLOA angled downward toward the lateral side (−2.7° ± 2.1°), consistent with previous studies [30,50]. Hutt et al. demonstrated that the actual joint line orientation was parallel to the floor on weight-bearing radiographs after kinematically aligned TKA, further advocating reconsideration of the established gold standard of TKA protocols [13]. Considering that the JLOA of healthy individuals was reported as 0.2° ± 1.1° in the study by Ji et al. [50] and 0.3° ± 2.0° in the study by Victor et al. [3], functionally aligned TKA appears to more closely reproduce the joint line orientation of the pre-arthritic knee compared to mechanically aligned TKA, suggesting a more balanced load distribution around the knee joint.

The weight-bearing position of the knee plays a crucial role in the long-term success of TKA, directly influencing polyethylene wear and the risk of implant loosening. A study by Tanaka et al. [31], involving 34 healthy participants with a mean age of 26.4 years, reported that their GA ratios were 51.4% and 50.4% during single-leg and double-leg standing positions, respectively, while their traditional MA ratios were 46.3% and 46.1%, respectively. Kikuchi et al. [51] recently demonstrated that GA provides a more accurate reflection of loading and exhibits a stronger correlation with knee adduction angular impulse compared to the traditional MA. Furthermore, Matsumoto et al. [52] showed that targeting the GA during TKA is feasible and may facilitate a physiological alignment closer to that of the native knee compared to other alignment techniques. Differences in coronal joint line configuration and weight-bearing position on hip-to-calcaneus radiographs may explain the superior clinical outcomes observed in the functionally aligned group compared to the mechanically aligned group. The functionally aligned group achieved higher FJS and greater ROM compared to the mechanically aligned group, in agreement with earlier findings [20,53,54,55,56]. However, despite the statistically significant improvements in PROMs, they did not exceed the minimal clinically important difference thresholds [57], suggesting a lack of clinical significance. Furthermore, although GA has been associated with a more balanced load distribution, its correlation with long-term outcomes and survival rates has yet to be fully validated. Therefore, a further prospective study with long-term follow-up is needed to determine whether these radiographic differences translate into better functional outcomes or implant survivorship.

This study had several limitations. First, it was a non-randomized, retrospective study utilizing data from a single institution. Second, the use of a robotic system could act as a confounding variable influencing the outcomes following TKA. However, functionally aligned TKA was performed using the robotic system to address the inaccuracies associated with manually performed functionally aligned procedures. Third, the knee joint line in the bipedal stance phase does not consider dynamic loading in the gait cycle. Therefore, we cannot conclude that the superior parallelism to the floor observed in the functionally aligned group remains consistent throughout the entire gait cycle. Fourth, relatively few patients participated in this study, which may have limited the determination of meaningful subgroup differences. As a result, we were unable to identify prognostic factors that influenced the benefits of FA-RTKA. A large-scale prospective study with long-term follow-up is needed to further clarify which patients benefit most from FA-RTKA. Finally, this study included the KOOS subscales to assess PROMs following TKA; however, these subscales do not assess detailed functional outcomes such as the rates of return to daily activities or pain during specific movements. Future studies are needed to incorporate such objective functional assessments to better elucidate patient benefits beyond standardized clinical scores.

## 5. Conclusions

Functionally aligned TKA showed enhanced joint line parallelism to the floor and a more neutral weight-bearing alignment in the GA than mechanically aligned TKA. These differences suggest a more balanced load distribution across the knee, potentially leading to improved clinical outcomes in the functionally aligned group.

## Figures and Tables

**Table 1:** Alignment targets in degrees (range) for different strategies between the groups.

Alignment	FA-RTKA	MA-CTKA
Coronal limits		
Femur	3° valgus to 3° varus	0°
Tibia	0° to 3° varus	0°
Sagittal limits		
Femur	0° to 5°	0° to 5°
Tibia	0° to 3°	0° to 3°
Axial		
Femur	sTEA ± 3	sTEA
Tibia	PCL – med 1/3 − lat 2/3 TT	PCL – med 1/3 − lat 2/3 TT
Hip-knee-ankle angle	0° to 3° varus	0°

**Table 2:** Demographic characteristics between the groups *.

	FA-RTKA (N = 50)	MA-CTKA (N = 50)	*p* Value ^†^
Age (year)	72.4 ± 6.7	71.3 ± 6.1	0.394
Female sex (no. of patients)	44 (88.0)	45 (90.0)	0.749
Body mass index (kg/m^2^)	26.1 ± 4.2	27.1 ± 3.4	0.200
ASA grade (no. of patients)			0.216
2	28 (56.0)	34 (68.0)	
3	22 (44.0)	16 (32.0)	
Side of operation (no. of patients)			0.841
Right	27 (54.0)	26 (52.0)	
Left	23 (46.0)	24 (48.0)	

* Values are given as the mean and the standard deviations or No. (%); ^†^ An independent *t*-test was used to analyze differences in age and body mass index. The chi-square or Fisher’s exact test was used to analyze the differences in sex, ASA grade, and the side of operation.

**Table 3:** Comparison of radiologic parameters between the groups *.

	FA-RTKA (N = 50)	MA-CTKA (N = 50)	*p* Value †
Preoperative findings			
HKA angle, varus (deg)	9.6 ± 3.7	9.3 ± 4.4	0.741
MPTA (deg)	85.3 ± 2.5	85.4 ± 2.2	0.829
LDFA (deg)	89.8 ± 2.4	89.4 ± 2.0	0.347
JLOA (deg)	−2.0 ± 2.6	−2.5 ± 2.3	0.134
Postoperative findings			
HKA angle, varus (deg)	2.0 ± 1.8	0.5 ± 2.2	**0.001**
MPTA (deg)	87.7 ± 1.3	89.7 ± 2.0	**<0.001**
LDFA (deg)	89.7 ± 1.7	90.5 ± 2.0	**0.030**
JLOA ‡ (deg)	0.6 ± 1.4	−2.7 ± 2.1	**<0.001**
Weight-bearing position in the knee joint §			
Traditional MA ratio	42.6 ± 7.6	48.3 ± 8.0	**<0.001**
GA ratio	48.8 ± 7.0	53.8 ± 8.8	**0.002**

* Values are given as the mean and the standard deviations; † An independent t-test was used to analyze differences. Bold values are significant (*p* < 0.05); ‡ A tibial joint line that angled down to the medial side was expressed as positive angle, while one that angled down to the lateral side was expressed as a negative angle; § The WBL ratios for MA and GA were determined by measuring lines passing through the knee center, with the medial edge designated as 0% and the lateral edge as 100%. The bold formatting highlights statistically significant differences.

**Table 4:** Comparison of clinical outcomes between the groups *.

	Preoperative				Postoperative		
	FA-RTKA (N = 50)	MA-CTKA (N = 50)	*p* Value ^†^		FA-RTKA (N = 50)	MA-CTK (N = 50)	*p* Value ^†^
Range of motion	121.1 ± 10.1	120.3 ± 12.9	0.730		131.1 ± 9.0	127.4 ± 8.2	**0.038**
KOOS							
Pain	42.7 ± 10.3	40.2 ± 10.8	0.233		84.9 ± 9.2	81.1 ± 13.0	0.091
Symptoms	48.3 ± 12.8	45.5 ± 14.6	0.303		85.4 ± 8.0	83.6 ± 10.9	0.360
Activities of daily living	41.7 ± 11.2	42.6 ± 9.0	0.664		79.3 ± 7.8	78.5 ± 10.1	0.674
Sports and recreation	23.7 ± 5.0	24.0 ± 5.7	0.781		26.9 ± 9.6	26.0 ± 6.0	0.576
Quality of life	21.2 ± 9.8	25.0 ± 11.3	0.100		64.4 ± 12.2	60.2 ± 14.8	0.120
FJS					80.1 ± 7.2	74.0 ± 9.5	**0.003**

* Values are given as the mean and the standard deviations; ^†^ The Mann−Whitney U test was used to analyze differences. Bold values are significant (*p* < 0.05); The bold formatting highlights statistically significant differences.

## Data Availability

All data are available from the corresponding author upon request.

## Abbreviations

The following abbreviations are used in this manuscript:

TKA	Total knee arthroplasty
MA	Mechanical axis
FA	Functional alignment
GA	Ground mechanical axis
HKA	Hip–knee–ankle
WBL	Weight-bearing line
MA	Mechanical axis
PS	Posterior stabilized
CT	Computed tomography
MPTA	Medial proximal tibial angle
LDFA	Lateral distal femoral angle
PROMs	Patient-reported outcome measures
KOOS	Knee Injury and Osteoarthritis Outcome Score
FJS	Forgotten Joint Score
ROM	Range of motion
SD	Standard deviation

## References

[1] Berend M.E., A Ritter M., Meding J.B., Faris P.M., Keating E.M., Redelman R., Faris G.W., E Davis K. (2004). Tibial component failure mechanisms in total knee arthroplasty. Clin. Orthop. Relat. Res..

[2] Longstaff L.M., Sloan K., Stamp N., Scaddan M., Beaver R. (2009). Good alignment after total knee arthroplasty leads to faster rehabilitation and better function. J. Arthroplast..

[3] Victor J.M., Bassens D., Bellemans J., Gürsu S., Dhollander A.A., Verdonk P.C. (2014). Constitutional varus does not affect joint line orientation in the coronal plane. Clin. Orthop. Relat. Res..

[4] Gu Y., Roth J.D., Howell S.M., Hull M.L. (2014). How Frequently Do Four Methods for Mechanically Aligning a Total Knee Arthroplasty Cause Collateral Ligament Imbalance and Change Alignment from Normal in White Patients? AAOS Exhibit Selection. J. Bone Jt. Surg. Am..

[5] Bourne R.B., Chesworth B.M., Davis A.M., Mahomed N.N., Charron K.D. (2010). Patient satisfaction after total knee arthroplasty: Who is satisfied and who is not?. Clin. Orthop. Relat. Res..

[6] Gunaratne R., Pratt D.N., Banda J., Fick D.P., Khan R.J.K., Robertson B.W. (2017). Patient Dissatisfaction Following Total Knee Arthroplasty: A Systematic Review of the Literature. J. Arthroplast..

[7] Nam D., Nunley R.M., Barrack R.L. (2014). Patient dissatisfaction following total knee replacement: A growing concern?. Bone Jt. J..

[8] Rivière C., Iranpour F., Auvinet E., Howell S., Vendittoli P.A., Cobb J., Parratte S. (2017). Alignment options for total knee arthroplasty: A systematic review. Orthop. Traumatol. Surg. Res..

[9] Maloney W.J., Barrack R.L., Berend K.R., Berry D.J., Della Valle C.J., Chen A.F., Dalury D.F., Haddad F.S., Lieberman J.R., Mayman D.J. (2023). Hot Topics and Current Controversies in Total Knee Arthroplasty. Instr. Course Lect..

[10] Howell S.M., Howell S.J., Kuznik K.T., Cohen J., Hull M.L. (2013). Does a kinematically aligned total knee arthroplasty restore function without failure regardless of alignment category?. Clin. Orthop. Relat. Res..

[11] Howell S.M., Shelton T.J., Hull M.L. (2018). Implant Survival and Function Ten Years After Kinematically Aligned Total Knee Arthroplasty. J. Arthroplast..

[12] Lee Y.S., Howell S.M., Won Y.Y., Lee O.S., Lee S.H., Vahedi H., Teo S.H. (2017). Kinematic alignment is a possible alternative to mechanical alignment in total knee arthroplasty. Knee Surg. Sports Traumatol. Arthrosc..

[13] Innocenti B., Bellemans J., Catani F. (2016). Deviations From Optimal Alignment in TKA: Is There a Biomechanical Difference Between Femoral or Tibial Component Alignment?. J. Arthroplast..

[14] Winnock de Grave P., Kellens J., Luyckx T., Tampere T., Lacaze F., Claeys K. (2022). Inverse Kinematic Alignment for Total Knee Arthroplasty. Orthop. Traumatol. Surg. Res..

[15] Almaawi A.M., Hutt J.R.B., Masse V., Lavigne M., Vendittoli P.A. (2017). The Impact of Mechanical and Restricted Kinematic Alignment on Knee Anatomy in Total Knee Arthroplasty. J. Arthroplast..

[16] Laforest G., Kostretzis L., Kiss M.O., Vendittoli P.A. (2022). Restricted kinematic alignment leads to uncompromised osseointegration of cementless total knee arthroplasty. Knee Surg. Sports Traumatol. Arthrosc..

[17] Bellemans J., Colyn W., Vandenneucker H., Victor J. (2012). The Chitranjan Ranawat award: Is neutral mechanical alignment normal for all patients? The concept of constitutional varus. Clin. Orthop. Relat. Res..

[18] Vanlommel L., Vanlommel J., Claes S., Bellemans J. (2013). Slight undercorrection following total knee arthroplasty results in superior clinical outcomes in varus knees. Knee Surg. Sports Traumatol. Arthrosc..

[19] Winnock de Grave P., Luyckx T., Claeys K., Tampere T., Kellens J., Müller J., Gunst P. (2022). Higher satisfaction after total knee arthroplasty using restricted inverse kinematic alignment compared to adjusted mechanical alignment. Knee Surg. Sports Traumatol. Arthrosc..

[20] Kayani B., Konan S., Tahmassebi J., Oussedik S., Moriarty P.D., Haddad F.S. (2020). A prospective double-blinded randomised control trial comparing robotic arm-assisted functionally aligned total knee arthroplasty versus robotic arm-assisted mechanically aligned total knee arthroplasty. Trials.

[21] Oussedik S., Abdel M.P., Victor J., Pagnano M.W., Haddad F.S. (2020). Alignment in total knee arthroplasty. Bone Jt. J..

[22] MacDessi S.J., Oussedik S., Abdel M.P., Victor J., Pagnano M.W., Haddad F.S. (2023). The language of knee alignment: Updated definitions and considerations for reporting outcomes in total knee arthroplasty. Bone Jt. J..

[23] Shatrov J., Foissey C., Kafelov M., Batailler C., Gunst S., Servien E., Lustig S. (2023). Functional Alignment Philosophy in Total Knee Arthroplasty-Rationale and Technique for the Valgus Morphotype Using an Image Based Robotic Platform and Individualized Planning. J. Pers. Med..

[24] Shatrov J., Battelier C., Sappey-Marinier E., Gunst S., Servien E., Lustig S. (2022). Functional Alignment Philosophy in Total Knee Arthroplasty—Rationale and technique for the varus morphotype using a CT based robotic platform and individualized planning. SICOT J..

[25] Van de Graaf V.A., Chen D.B., Allom R.J., Wood J.A., MacDessi S.J. (2023). Functional alignment in total knee arthroplasty best achieves balanced gaps and minimal bone resections: An analysis comparing mechanical, kinematic and functional alignment strategies. Knee Surg. Sports Traumatol. Arthrosc..

[26] Guichet J.M., Javed A., Russell J., Saleh M. (2003). Effect of the foot on the mechanical alignment of the lower limbs. Clin. Orthop. Relat. Res..

[27] Desai S.S., Shetty G.M., Song H.R., Lee S.H., Kim T.Y., Hur C.Y. (2007). Effect of foot deformity on conventional mechanical axis deviation and ground mechanical axis deviation during single leg stance and two leg stance in genu varum. Knee.

[28] Haraguchi N., Ota K., Tsunoda N., Seike K., Kanetake Y., Tsutaya A. (2015). Weight-bearing-line analysis in supramalleolar osteotomy for varus-type osteoarthritis of the ankle. J. Bone Jt. Surg. Am..

[29] Cooke T.D., Pichora D., Siu D., Scudamore R.A., Bryant J.T. (1989). Surgical implications of varus deformity of the knee with obliquity of joint surfaces. J. Bone Jt. Surg. Br..

[30] Matsumoto T., Takayama K., Ishida K., Hayashi S., Hashimoto S., Kuroda R. (2017). Radiological and clinical comparison of kinematically versus mechanically aligned total knee arthroplasty. Bone Jt. J..

[31] Tanaka T., Takayama K., Hashimoto S., Kanzaki N., Hayashi S., Kuroda R., Matsumoto T. (2017). Radiographic analysis of the lower limbs using the hip-calcaneus line in healthy individuals and in patients with varus knee osteoarthritis. Knee.

[32] Gustke K.A., Simon P. (2024). A Restricted Functional Balancing Technique for Total Knee Arthroplasty With a Varus Deformity: Does a Medial Soft-Tissue Release Result in a Worse Outcome?. J. Arthroplast..

[33] Yang H.Y., Cheon J.H., Kang S.J., Seon J.K. (2024). Effect of tibia-first, restricted functional alignment technique on gap width changes, and component positioning in robotic arm-assisted total knee arthroplasty. Knee Surg. Sports Traumatol. Arthrosc..

[34] Hutt J., Massé V., Lavigne M., Vendittoli P.A. (2016). Functional joint line obliquity after kinematic total knee arthroplasty. Int. Orthop..

[35] Roos E.M., Roos H.P., Lohmander L.S., Ekdahl C., Beynnon B.D. (1998). Knee Injury and Osteoarthritis Outcome Score (KOOS)--development of a self-administered outcome measure. J. Orthop. Sports Phys. Ther..

[36] Behrend H., Giesinger K., Giesinger J.M., Kuster M.S. (2012). The “forgotten joint” as the ultimate goal in joint arthroplasty: Validation of a new patient-reported outcome measure. J. Arthroplast..

[37] Kafelov M., Batailler C., Shatrov J., Al-Jufaili J., Farhat J., Servien E., Lustig S. (2023). Functional positioning principles for image-based robotic-assisted TKA achieved a higher Forgotten Joint Score at 1 year compared to conventional TKA with restricted kinematic alignment. Knee Surg. Sports Traumatol. Arthrosc..

[38] Howell S.M., Papadopoulos S., Kuznik K., Ghaly L.R., Hull M.L. (2015). Does varus alignment adversely affect implant survival and function six years after kinematically aligned total knee arthroplasty?. Int. Orthop..

[39] Kayani B., Konan S., Tahmassebi J., Pietrzak J.R.T., Haddad F.S. (2018). Robotic-arm assisted total knee arthroplasty is associated with improved early functional recovery and reduced time to hospital discharge compared with conventional jig-based total knee arthroplasty: A prospective cohort study. Bone Jt. J..

[40] Burssens A.B., Buedts K., Barg A., Vluggen E., Demey P., Saltzman C.L., Victor J.M. (2020). Is Lower-limb Alignment Associated with Hindfoot Deformity in the Coronal Plane? A Weightbearing CT Analysis. Clin. Orthop. Relat. Res..

[41] Dagneaux L., Dufrenot M., Bernasconi A., Bedard N.A., de Cesar Netto C., Lintz F. (2020). Three-Dimensional Biometrics to Correlate Hindfoot and Knee Coronal Alignments Using Modern Weightbearing Imaging. Foot Ankle Int..

[42] Ritter M.A., Davis K.E., Meding J.B., Pierson J.L., Berend M.E., Malinzak R.A. (2011). The effect of alignment and BMI on failure of total knee replacement. J. Bone Jt. Surg. Am..

[43] Insall J.N., Binazzi R., Soudry M., Mestriner L.A. (1985). Total knee arthroplasty. Clin. Orthop. Relat. Res..

[44] Parratte S., Pagnano M.W., Trousdale R.T., Berry D.J. (2010). Effect of postoperative mechanical axis alignment on the fifteen-year survival of modern, cemented total knee replacements. J. Bone Jt. Surg. Am..

[45] MacDessi S.J., Griffiths-Jones W., Chen D.B., Griffiths-Jones S., Wood J.A., Diwan A.D., Harris I.A. (2020). Restoring the constitutional alignment with a restrictive kinematic protocol improves quantitative soft-tissue balance in total knee arthroplasty: A randomized controlled trial. Bone Jt. J..

[46] Koh I.J., Park I.J., Lin C.C., Patel N.A., Chalmers C.E., Maniglio M., McGarry M.H., Lee T.Q. (2019). Kinematically aligned total knee arthroplasty reproduces native patellofemoral biomechanics during deep knee flexion. Knee Surg. Sports Traumatol. Arthrosc..

[47] Lovejoy C.O. (2007). The natural history of human gait and posture. Part 3. The knee. Gait Posture.

[48] Howell S.M., Hodapp E.E., Vernace J.V., Hull M.L., Meade T.D. (2013). Are undesirable contact kinematics minimized after kinematically aligned total knee arthroplasty? An intersurgeon analysis of consecutive patients. Knee Surg. Sports Traumatol. Arthrosc..

[49] Howell S.M., Papadopoulos S., Kuznik K.T., Hull M.L. (2013). Accurate alignment and high function after kinematically aligned TKA performed with generic instruments. Knee Surg. Sports Traumatol. Arthrosc..

[50] Ji H.M., Han J., Jin D.S., Seo H., Won Y.Y. (2016). Kinematically aligned TKA can align knee joint line to horizontal. Knee Surg. Sports Traumatol. Arthrosc..

[51] Kikuchi N., Kanamori A., Kadone H., Okuno K., Hyodo K., Yamazaki M. (2022). Radiographic analysis using the hip-to-calcaneus line and its association with lower limb joint kinetics in varus knee osteoarthritis. Knee.

[52] Matsumoto T., Nakano N., Ishida K., Maeda T., Tachibana S., Kuroda Y., Hayashi S., Matsushita T., Kuroda R. (2023). Targeting the neutral hip-to-calcaneus axis in kinematically aligned total knee arthroplasty is feasible with fewer alignment outliers for varus osteoarthritic patients. Knee Surg. Sports Traumatol. Arthrosc..

[53] Zambianchi F., Bazzan G., Marcovigi A., Pavesi M., Illuminati A., Ensini A., Catani F. (2021). Joint line is restored in robotic-arm-assisted total knee arthroplasty performed with a tibia-based functional alignment. Arch. Orthop. Trauma. Surg..

[54] Kayani B., Fontalis A., Haddad I.C., Donovan C., Rajput V., Haddad F.S. (2023). Robotic-arm assisted total knee arthroplasty is associated with comparable functional outcomes but improved forgotten joint scores compared with conventional manual total knee arthroplasty at five-year follow-up. Knee Surg. Sports Traumatol. Arthrosc..

[55] Ali M., Kamson A., Yoo C., Singh I., Ferguson C., Dahl R. (2023). Early Superior Clinical Outcomes in Robotic-Assisted TKA Compared to Conventional TKA in the Same Patient: A Comparative Analysis. J. Knee Surg..

[56] Liow M.H.L., Goh G.S., Wong M.K., Chin P.L., Tay D.K., Yeo S.J. (2017). Robotic-assisted total knee arthroplasty may lead to improvement in quality-of-life measures: A 2-year follow-up of a prospective randomized trial. Knee Surg. Sports Traumatol. Arthrosc..

[57] Robinson P.G., MacDonald D.J., Macpherson G.J., Patton J.T., Clement N.D. (2021). Changes and thresholds in the Forgotten Joint Score after total hip arthroplasty: Minimal clinically important difference, minimal important and detectable changes, and patient-acceptable symptom state. Bone Jt. J..

